# RNAi-mediated down-regulation of the expression of *OsFAD2-1*: effect on lipid accumulation and expression of lipid biosynthetic genes in the rice grain

**DOI:** 10.1186/s12870-016-0881-6

**Published:** 2016-08-31

**Authors:** Gopal Ji Tiwari, Qing Liu, Pushkar Shreshtha, Zhongyi Li, Sadequr Rahman

**Affiliations:** 1 School of Science, Monash University Malaysia, 46150 Bandar Sunway, Selangor, Malaysia; 2 Monash University Malaysia Genomics Facility, 46150 Bandar Sunway, Selangor, Malaysia; 3 CSIRO Agriculture & Food, PO Box 1600, Canberra, ACT 2601 Australia

**Keywords:** Rice bran oil, Triacylglycerol, Oleic acid, FAD2, Transcriptome

## Abstract

**Background:**

The bran from polished rice grains can be used to produce rice bran oil (RBO). High oleic (HO) RBO has been generated previously through RNAi down-regulation of *OsFAD2-1*. HO-RBO has higher oxidative stability and could be directly used in the food industry without hydrogenation, and is hence free of *trans* fatty acids. However, relative to a classic oilseed, lipid metabolism in the rice grain is poorly studied and the genetic alteration in the novel HO genotype remains unexplored.

**Results:**

Here, we have undertaken further analysis of role of *OsFAD2-1* in the developing rice grain. The use of Illumina-based NGS transcriptomics analysis of developing rice grain reveals that knockdown of *Os-FAD2-1* gene expression was accompanied by the down regulation of the expression of a number of key genes in the lipid biosynthesis pathway in the HO rice line. A slightly higher level of oil accumulation was also observed in the HO-RBO.

**Conclusion:**

Prominent among the down regulated genes were those that coded for FatA, LACS, SAD2, SAD5, caleosin and steroleosin. It may be possible to further increase the oleic acid content in rice oil by altering the expression of the lipid biosynthetic genes that are affected in the HO line.

**Electronic supplementary material:**

The online version of this article (doi:10.1186/s12870-016-0881-6) contains supplementary material, which is available to authorized users.

## Background

Rice is one of the most important crops for mankind as it provides nearly half of the world’s population a source of dietary energy [1]. Apart from starch, rice grains contain a small proportion of lipids (1–4 % of the grain) located mostly in the bran. Rice bran oil (RBO) is extracted from rice bran as a by-product of milling and is commercially available as a food grade vegetable oil [2, 3]. Triacylglycerols (TAGs) make up about 85 % of the total lipids in RBO, followed by phospholipids (~6.5 %) and free fatty acids (~4.5 %) [4]. RBO is also rich in compounds such as oryzanol and tocotrienes having antioxidant and cholesterol–reducing activities [5–8]. TAGs inRBO are composed of three main fatty acids: palmitic acid, oleic acid and linoleic acid. The relative content of palmitic (15–20 %), oleic (36–48 %) and linoleic acids (30–38 %) depends on the cultivar and environment [9, 10].

Linoleic acid can undergo non-enzymatic oxidation because of the presence of the two reactive double bonds in the molecule [11, 12] which reduces the shelf-life of RBO and leads to wastage of 60–70 % of RBO [6, 13]. Therefore, partial hydrogenation has often been used to enhance the oxidative stability of RBO, resulting in nutritionally undesirable *trans* fatty acids as a by-product. *Trans* fatty acids have been found to increase the risk of cardiovascular diseases and have been prohibited in foods in an increasing number of countries in the world [14–17]. On the other hand, oleic acid is both oxidatively stable and nutritionally desirable, hence favored for direct food applications without partial hydrogenation.

The microsomal enzyme ∆12 fatty acid desaturase (FAD2) converts oleic acid into linoleic acid while associated with phosphatidylcholine in the endoplasmic reticulum (ER). A total of 18 desaturase genes have been annotated in rice genome, among which are the four *FAD2* genes investigated by Zaplin et al*.* [18]. These were termed *OsFAD2*–*1*, –*2,* –*3* and –*4*. Among these four genes, the expression of *OsFAD2*–*1* was reduced by RNA interference (RNAi) suppression which resulted in an increase in the proportion of oleic acid and a reduction of the proportions of linoleic and palmitic acids in T_3_ grains. Our previous results suggested that the *OsFAD2–1*gene was an effective target for raising oleic acid levels at the expense of the oxidatively unstable linoleic acid and the cholesterol-raising palmitic acid [18].

Most reports of genetic modification and characterisation of oil accumulation in plants have so far been carried out in Arabidopsis and classic dicot oilseed crops and focused mainly on trait development [19–24]. We have therefore decided to investigate further the role of the *OsFAD2-1* gene in the rice grain. The comparative analysis of lipid fractions in wild type (WT) and HO-RBO was carried out. We also describe the use of Illumina-based NGS transcriptomic analysis on the same selected HO rice line to study the effect of RNAi down-regulation of *OsFAD2-1* on the grain transcriptome, especially on other genes that are involved in lipid biosynthesis and turnover. Preliminary qPCR experiments confirmed the transcriptomic results for some of the selected genes. In this paper we also show that the down-regulation of *OsFAD2-1*with a seed-specific promoter to produce HO rice line was not associated with compromised oil accumulation in the grain, but rather a modest increase.

## Results and discussion

### Analysis of lipid composition in rice grains from HO rice line and its null segregant

Total lipids were analysed from the HO rice grains. These grains were from the homozygous transgenic line containing the *OsFAD2-1* RNAi construct that was used for transcriptomics analysis. The total lipids in the HO rice grain were composed of 55.0 % oleic acid, 19.8 % linoleic acid and 16.8 % palmitic acid, whereas the grains from a null segregant (a sister line derived from the same original transformation event that does not contain the *OsFAD2-1 RNAi* construct) comprised 32.3 % oleic acid, 40.7 % linoleic acid and 18.6 % palmitic acid (Table 1). The oleic acid content from HO rice line was significantly higher than that from its null segregant (*p* < 0.05). Similar changes were also observed in TAG and phosphatidylcholine (PC) pools, however, there were somewhat different fatty acid compositional profiles for polar lipids, such as the phosphatidylethanolamine (PE) and phosphatidylcholine (PC) pools. The overall results are in broad agreement with the results from Zaplin et al*.* [18] from an earlier generation of this material (Additional file 1).

**Table 1:** Fatty acid composition of rice grains of *OsFAD2-1 RNAi* line and its null segregant line

	Total lipids		Triacylglycerols		Polar lipid pool		Free fatty acids		PC		PE	
	Control	Fad2	Control	Fad2	Control	Fad2	Control	Fad2	Control	Fad2	Control	Fad2
CI4:0	0.6 ± 0.0	0.3 ± 0.0	0.5 ± 0.0	0.3 ± 0.0	2.5 ± 0.1	1.2 ± 0.1	1.6 ± 0.0	0.9 ± 0.1	1.1	0.6	1.6	1.2
CI6:0	18.6 ± 0.2	16.8 ± 0.4	18.4 ± 0.1	16.3 ± 0.3	26.7 ± 1.0	25.2 ± 0.8	18.7 ± 0.6	22.2 ± 0.6	21.0	17.8	25.8	24.1
CI6:1	0.3 ± 0.0	0.3 ± 0.0	0.3 ± 0.0	0.3 ± 0.0	0.2 ± 0.0	0.3 ± 0.0	0.2 ± 0.0	0.3 ± 0.0	0.3	0.3	0.3	0.3
CI8.0	2.4 ± 0.0	2.6 ± 0.1	2.4 ± 0.1	2.6 ± 0.1	1.8 ± 0.1	2.1 ± 0.1	3.0 ± 0.1	3.8 ± 0.2	1.5	1.4	2.2	19
08.1	32.3 ± 0.4	55.0 ± 0.7	33.8 ± 0.3	56.2 ± 0.7	24.1 ± 1.0	43.9 ± 14	11.8 ± 0.3	45.4 ± 0.9	38.8	55.4	25.4	41.8
C18:ld11	1.0 ± 0.0	1.1 ± 0.0	1.0 ± 0.0	1.1 ± 0.0	1.1 ± 0.0	1.2 ± 0.0	0.6 ± 00	0.8 ± 0.0	1.4	1.4	1.3	1.3
CI8.2	40.7 ± 0.4	19.8 ± 0.7	40.2 ± 0.5	19.8 ± 0.6	36.6 ± 0.4	20.0 ± 0.6	58.9 ± 0.6	21.4 ± 1.3	33.2	20.4	40.3	26.2
CI8:3n3	1.7 ± 0.1	1.5 ± 0.1	1.7 ± 0.1	1.4 ± 0.1	1.5 ± 0.2	1.5 ± 0.1	2.5 ± 0.0	2.4 ± 0.2	1.4	1.2	1.3	1.1
C20:0	0.7 ± 0.0	0.8 ± 0.0	0.7 ± 0.0	0.8 ± 0.0	0.3 ± 0.0	0.3 ± 0.0	0.3 ± 00	0.4 ± 0.0	0.2	0.2	0.2	0.3
C20:1d11	0.4 ± 0.0	0.6 0.0	0.4 ± 0.0	0.6 ± 0.0	0.1 ± 0.0	0.2 ± 0.0	0.1 ± 0.1	02 ± 0.0	0.2	0.2	0.1	0.2
C22:0	0.4 ± 0.0	0.4 ± 0.0	0.2 ± 0.0	0.2 ± 0.0	1.3 ± 0.1	1.1 ± 0.1	0.5 ± 0.0	0.5 ± 0.0	0.3	0.3	0.4	0.4
C24:0	0.8 ± 0.0	0.8 ± 0.0	0.4 ± 0.0	0.4 ± 0.0	3.7 ± 0.3	3.2 ± 0.3	1.9 ± 0.1	1.8 ± 02	0.7	0.7	1.2	1.3
% oil/wt	2.6 ± 0.1	2.9 ± 0.1	1.8 ± 0.1	2.1 ± 0.1	0.21 ± 0.01	0.23 ± 0.00	0.07 ± 0.00	0.08 ± 0.01	0.06	0.08	0.02	0.02

Control: represents grains from null segregant; Fad2: represents grains from *OsFAD2-1*RNAi line; numbers represent mean ± SE in percentage (%); Mean Values are from three repeat analyses of lipid samples which were extracted separately from three independent grain samples

Grains from *OsFAD2-1* RNAi line contained higher levels of total lipids (2.9 % by dry weight) compared to 2.6 % in its null segregant (*p* < 0.05), which was reflected by the significant increases in both TAG and polar lipids.

### Transcriptome analysis of rice immature endosperms from HO rice line and its null segregant

RNAseq reads from three developmental stages of endosperm of both the HO rice line and its null segregant were mapped against the reference rice genome (cultivar *Nipponbare*) [25] to generate the mapped contigs as summarised in Table 2. In total, 1.5–9 million of contigs per sample were assembled which included approximately 80–94 % counted contigs for use in further analysis, and 6–20 % un-counted contigs, defined as the total number of fragments after sequencing which could not be mapped, either as intact or as broken pairs. Among the counted contigs, 75–86 % were unique, and 3–10%were non-specific contigs, defined as the reads which have multiple equally good alignments to the reference and therefore have to be excluded from the RNA-seq analysis.

**Table 2:** Mapped contig results of RNA-Seq reads from null segregant (NG) and *OsFAD2-1*RNAi rice lines at three grain developmental stages

Contigs	Null segregant			*Os-FAD2*-1 RNAi		
	Sample1	Sample 2	Sample 3	Sample 1	Sample 2	Sample 3
10 DAA						
Counted contigs	1,474,350	2,451,049	912,841	2,305,750	7,974,195	3,179,294
Unique contigs	1,403,705	2,334,799	858,280	2,090,665	7,406,550	3,050,886
Non-S contigs	70,645	116,250	54,561	215,085	567,645	128,408
Un-C contigs	380,469	691,998	678,922	221,969	1,119,121	590,977
Total contigs	1,854,819	3,143,047	1,591,763	2,527,719	9,093,316	3,770,271
Counted contigs (%)	79.49	77.98	57.35	91.22	87.69	84.33
Unique contigs (%)	75.68	74.28	53.92	82.71	81.45	80.92
Non-S contigs (%)	3.81	3.70	3.43	8.51	6.24	3.41
Un-C contigs (%)	20.51	22.02	42.65	8.78	12.31	15.67
15 DAA						
Counted contigs	1,721,045	4,038,637	6,507,485	1,260,698	4,102,787	5,184,375
Unique contigs	1,580,034	3,759,033	5,944,747	1,211,621	3,877,771	4,889,384
Non-S contigs	141,011	279,604	562,738	49,077	225,016	294,991
Un-C contigs	210,716	347,568	496,403	385,614	1,123,069	436,145
Total contigs	1,931,761	4,386,205	7,003,888	1,646,312	5,225,856	5,620,520
Counted contigs (%)	89.09	92.08	92.91	76.58	78.51	92.24
Unique contigs (%)	81.79	85.70	84.88	73.60	74.20	86.99
Non-S contigs (%)	7.30	6.37	8.03	2.98	4.31	5.25
Un-C contigs (%)	10.91	7.92	7.09	23.42	21.49	7.76
20 DAA						
Counted contigs	2,945,375	1,943,916	1,348,074	3,914,475	791,645	3,627,328
Unique contigs	2,797,599	1,778,024	1,212,290	3,446,816	734,727	3,386,969
Non-S contigs	147,776	165,892	135,784	467,659	56,918	240,359
Un-C contigs	447,772	250,284	168,761	441,185	464,829	492,027
Total contigs	3,393,097	2,194,200	1,516,835	4,355,660	1,256,474	4,119,355
Counted contigs (%)	89.09	88.59	88.87	89.87	63.01	88.06
Unique contigs (%)	86.80	81.03	79.92	79.13	58.48	82.22
Non-S contigs (%)	82.45	7.56	8.95	10.74	4.53	5.83
Un-C contigs (%)	4.36	11.41	11.13	10.13	36.99	11.94

*Non-S contigs-* Non-specific contigs; *Un C contigs*-Un-counted contigs

The genes analysed could be grouped broadly into four categories: genes known to be involved in fatty acid biosynthesis and degradation, genes involved in TAG metabolism, transcriptional factors and other genes found to be affected (Additional file 2 and Additional file 3). A total of 55,801 different gene transcripts were detected in the overall analyses out of which 1,617 (2.9 %) genes at 10 days after anthesis (DAA), 1,175 (2.1 %) genes at 15 DAA and 626 (1.12 %) genes at 20 DAA showed significant differences in expression between the null segregant and the HO rice line.

### Expression of genes involved in fatty acid biosynthesis and degradation

*De novo* fatty acid biosynthesis occurs primarily in plastids, although it also occurs in the mitochondrion to a much lesser extent [26, 27]. The first addition of a malonyl group to an acetyl group is catalysed by KASIII, while the subsequent acyl chain elongation up to C16 and the final two-carbon extension to form C18 fatty acid while associated with acyl carrier protein (ACP) are catalysed by KASI and KASII, respectively (Additional file 4: Table S1). None of the putative transcripts for *KAS* genes were affected by the RNAi down-regulation of *OsFAD2-1* gene (LOC_Os02g48560) (Additional file 5).

**Fig. 1:**
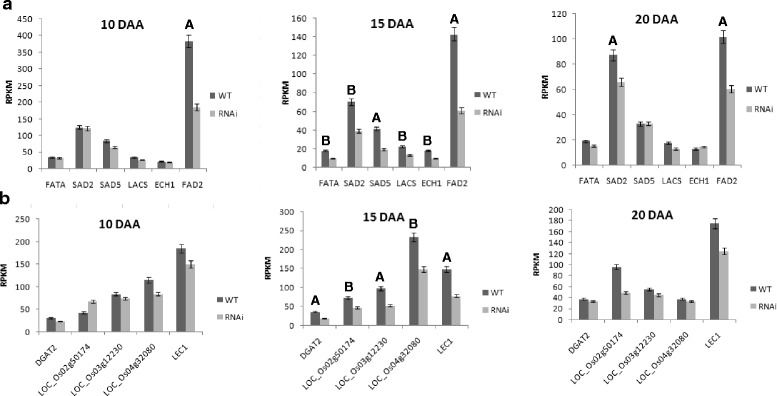
Differential transcript expression of genes involved in rice (**a**) fatty acid biosynthesis and (**b**) lipid metabolism between the null segregates and *OsFAD2-1* RNAi lines. Developing stages of immature endosperm and gene types are indicated above each figure, the values on the y-axis represent RPKM, and gene locus and their names are labelled underneath. Data analysed using CLC-Bio Genomic Workbench. Baggerley’s test was conducted for analysing genes between the null segregates (WT or NG) and *OsFAD2-1* RNAi (RNAi) lines. The letter **a**: indicates significant results at p value ≤ 0.01, the letter **b**: indicates significant results at 0.01 ≥ p value ≤ 0.05. FAT: Acyl-ACP thioesterase A, SAD: stearoyl-ACP desaturases, LACS: Long-chain acyl-CoA synthetase, ECH1: enoyl-CoA hydratase 1, FAD: fatty acid desaturases, DGAT:acyl-CoA:DAG acyltransferase, LEC1: Leafycotyledon1

Termination of fatty acid elongation in plastids is catalysed by acyl-ACP thioesterase enzymes (Fat), 25 unigenes of which have been annotated in the Rice Genome project [25]. Among them *FatA* and *FatB* are represented by LOC_Os09g32760 and LOC_Os06g05130, respectively. FatA preferentially catalyses the cleavage of the thioester bond of oleyl-ACP, and is also regarded as one of the key enzymes responsible for oleic acid concentration in oil and FatB has substrate preference forC16 - C18 saturated fatty acids [28]. Expression of *FatA* was found significantly reduced at 15 DAA by -1.62 fold (*p* = 0.04) equivalent to -0.91 log 2 fold (Table 3). This is in contrast to the transcript abundance of *FatB* that was not affected in the RNAi-*OsFAD2-1*line, compared to the null segregant control, in all three developmental stages analysed (Fig. 1a). Significant differences in the expression levels of *FatA* and *FatB* were not observed at 10 and 20 DAA.

**Table 3:** Differential expression of genes in the metabolism of Fatty acid and TAG biosynthesis

Feature ID	Gene abbreviation	DAA	Weighted proportions fold change	*P*-value	RNAi/WT mean fold change	RNAi/WT mean log2 fold change
LOC_Os09g32760	FATA	10	−1.1	0.54	0.93	−0.11
LOC_Os09g32760	FATA	15	−1.62	*0.04	0.53	−0.91
LOC_Os09g32760	FATA	20	−1.28	0.22	0.80	−0.32
LOC_Os01g69080	SAD2	10	−1.04	0.76	0.98	−0.03
LOC_Os01g69080	SAD2	15	−1.57	*0.02	0.55	−0.85
LOC_Os01g69080	SAD2	20	−1.35	*0.01	0.75	−0.41
LOC_Os04g31070	SAD5	10	−1.31	0.2	0.78	−0.37
LOC_Os04g31070	SAD5	15	−1.88	*2.17E-4	0.46	−1.12
LOC_Os04g31070	SAD5	20	−1.01	0.92	1.01	0.01
LOC_Os05g25310	LACS	10	−1.3	0.13	0.78	−0.36
LOC_Os05g25310	LACS	15	−1.45	*0.04	0.59	−0.76
LOC_Os05g25310	LACS	20	−1.37	0.23	0.74	−0.43
LOC_Os01g70090	ECH1	10	−1.11	0.56	0.91	−0.13
LOC_Os01g70090	ECH1	15	−1.64	^a^0.03	0.53	−0.93
LOC_Os01g70090	ECH1	20	1.06	0.79	1.11	0.15
LOC_Os02g48560	FAD2	10	−2.1	*2.02E-3	0.48	−1.05
LOC_Os02g48560	FAD2	15	−2.05	*9.15E-6	0.43	−1.22
LOC_Os02g48560	FAD2	20	−1.77	*0.04	0.59	−0.75
LOC_Os06g22080	DGAT2	10	−1.31	0.45	0.76	−0.39
LOC_Os06g22080	DGAT2	15	−1.71	*7.73E-3	0.51	−0.98
LOC_Os06g22080	DGAT2	20	−1.16	0.28	0.89	−0.17
LOC_Os02g50174	Caleosin	10	1.52	0.27	1.56	0.64
LOC_Os02g50174	Caleosin	15	−1.33	*0.04	0.65	−0.63
LOC_Os02g50174	Caleosin	20	−1.97	*5.02E-3	0.51	−0.97
LOC_Os03g12230	Caleosin	10	−1.14	0.42	0.88	−0.18
LOC_Os03g12230	Caleosin	15	−1.58	*6.60E-3	0.55	−0.86
LOC_Os03g12230	Caleosin	20	−1.27	0.5	0.81	−0.31
LOC_Os04g32080	STEROLEOSIN	10	−1.36	0.42	0.73	−0.45
LOC_Os04g32080	STEROLEOSIN	15	−1.36	*0.03	0.64	−0.65
LOC_Os06g22080	STEROLEOSIN	20	−1.16	0.28	0.89	−0.17
LOC_Os02g49410	LEC1	10	−1.23	0.42	0.81	−0.30
LOC_Os02g49410	LEC1	15	−1.66	*3.91E-3	0.53	−0.92
LOC_Os02g49410	LEC1	20	−1.44	0.12	0.71	−0.49

*represents significant *p*-values

The first desaturation step of a saturated fatty acid occurs in the plastids, catalysed by stearoyl-ACP desaturase (SAD). SAD is a soluble plastidial enzyme that introduces the first double bond into stearic acid and to a lesser extent palmitic acid to form oleic acid and palmitoleic acid, respectively. LOC_Os01g69080annotated as *SAD2*gene was highly expressed in rice grains at 10 DAA. In comparison to the null segregant, the expression level of *SAD2* was reduced by −1.6 and −1.35 fold in the HO rice grains at 15 DAA (*p* = 0.02) and 20 DAA (*p* = 0.01) respectively, while no significant difference was observed at 10 DAA (Table 3, Fig. 1a). *SAD5* (LOC_Os04g31070) expression was also found to be down regulated at 15 DAA by -1.88 fold (*p* = 2.17E-4) and −1.12 log2 fold change (Table 3, Fig. 1a). No significant change in expression was found in other unigenes annotated for encoding SAD in the HO line compared to null segregant (Additional file 2).

The nucleotide sequence alignment match between either of *SAD2* or *SAD5* and *OsFAD2-1* is generally low and stretches of 20 nucleotide DNA sequences with significant identity were not found. It is therefore unlikely that the decrease in expression level of *SAD* genes in HO line was due to cross silencing. As SAD is an upstream fatty acid desaturase of FAD2, it is tempting to assume that the reduction in the expression of *OsFAD2-1* leading to the build-up of oleic acid may have a feedback effect that leads to the down regulation of SAD expression which is responsible for oleic acid production.

Oleic acid could be further modified by FAD2 in endoplasmic reticulum (ER) through the eukaryotic pathway or by FAD6 in plastids via the prokaryotic pathway. In the previous study [18], four genes in the rice genome were putatively identified as *FAD2* that are present in the eukaryotic pathway, LOC_Os02g48560 (*OsFAD2-1*), LOC_Os07g23430 (*OsFAD2-2*), LOC_Os07g23410 (*OsFAD2-3*) and LOC_Os07g23390 (*OsFAD2-4*). Transcriptome analysis showed that the expression patterns of all the four *OsFAD2* genes were consistent with the previous data of Zaplin et al. [18] and the analysis of publicly available transcriptome data (Additional file 6: Table S2). The analysis of transcriptome data described in this paper showed that, only *OsFAD2-1* transcripts were found in all three grain developmental stages (10, 15 and 20 DAA) (Table 4). The highest expression level of *OsFAD2-1* was found in the early developmental stage in the null segregant line and it declined as the grains developed. Such a finding is consistent with Wang et al. [29] who found that in sesame most of the genes related to lipid biosynthesis were highly expressed at early stage of seed development, which is at 10 DAA. This may suggest that the biosynthesis of polyunsaturated fatty acids is initiated at a rather early stage of grain development. Such a factor needs to be considered for the choice of promoter that drives the hairpin expression cassette of the *OsFAD2-1* sequence in RNAi construct. The HO rice line was generated by using a storage protein promoter, Bx17, which becomes most active from the mid-stage of endosperm development onwards [18]. It is tempting to assume that further enhancement of oleic acid accumulation above that observed in the current transgenic lines is possible when an alternative grain- or bran- specific promoter that is active from early grain development is employed.

**Table 4:** Expressionlevels of four *FAD2* genes in a null segregant (NG) and an *OsFAD2-1*RNAi line at 10, 15 and 20 DAA developmental stages

DAA	Gene	Rice Genome Annotation Project locus ID	NG 1 (RPKM)	NG2 (RPKM)	NG3 (RPKM)	NG (RPKM mean)	*OsFAD2-1*RNAi 1 (RPKM)	*OsFAD2-1*RNAi 2 (RPKM)	*OsFAD2-1 RNAi 3 (RPKM)*	*OsFAD2-1*RNAi (RPKM mean)
10	*OsFAD2–1*	LOC_Os02g48560	296.6	371.57	482.81	383.66	188.21	210.85	158.01	185.69
15	*OsFAD2–1*	LOC_Os02g48560	134.18	133.69	160.53	142.8	34.8	89.31	59.16	61.09
20	*OsFAD2–1*	LOC_Os02g48560	89.67	89.96	128.16	101.93	54.63	55.79	70.9	60.44
10	*OsFAD2–2*	LOC_Os07g23430	0	0	0	0	0	0	0	0
15	*OsFAD2–2*	LOC_Os07g23430	0	0	0	0	0	0	0	0
20	*OsFAD2–2*	LOC_Os07g23430	0	0	0	0	0.13	0	1.3	0.48
10	*OsFAD2–3*	LOC_Os07g23410	0	0	0.15	0.53	0.15	0.5	0	0.23
15	*OsFAD2–3*	LOC_Os07g23410	0.19	0.06	0	0	0	0	0	0
20	*OsFAD2–3*	LOC_Os07g23410	0.46	0.15	0.31	0	0.31	0	0	0.10
10	*OsFAD2–4*	LOC_Os07g23390	0	0	0	0	0	0	0	0
15	*OsFAD2–4*	LOC_Os07g23390	0	0.22	0	0.07	1.18	0.36	0	0.51
20	*OsFAD2–4*	LOC_Os07g23390	0	0	0	0	0	0.82	1.87	0.90

*DAA-*days after anthesis

The expression of *OsFAD2-1* in the HO rice lines was significantly down regulated in all the three developmental stages examined, with the most marked reduction by -2.05 fold (*p* = 9.15E-6) and -1.22 log2 fold at 15 DAA (Table 3, Fig. 1a). This is anticipated because *OsFAD2-1*was specifically targeted by RNAi mediated gene silencing. However, the down-regulation of *OsFAD2-1* expression did not result in detectable level of alteration in the already very low expression of *OsFAD2-2, -3, -4* genes at 10, 15 and 20 DAA stages.

### Effect on long chain fatty acyl-CoA synthetases (LACS) genes

**Fig. 2:**
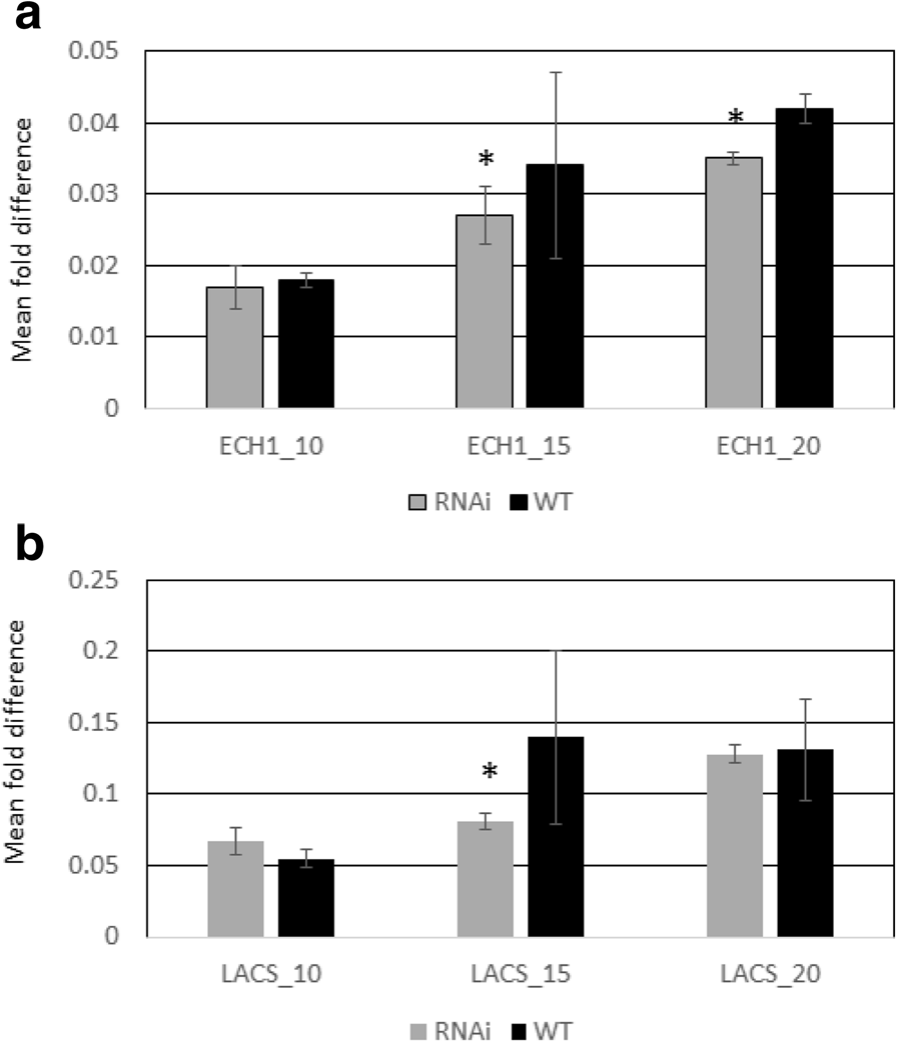
Level of ECH1 and LACS transcripts of two *OsFAD2-1* RNAi lines (RNAi) and two null segregants (WT) at three different developmental stages. The ∆∆CT method [55] was used to determine the expression of the ECH1 (**a**) and LACS (**b**) gene transcripts normalised to the α-tubulin housekeeping gene from qRT-PCR data to produce a mean fold difference. Error bars are one standard error (s.e). ECH1_10: ECH1 gene at 10 DPA; ECH1_15: ECH1 gene at 15DAA; ECH1_20: ECH1 gene at 20 DAA; LACS_10: LACS gene at 10 DAA; LACS_15: LACS gene at 15 DAA; LACS_20: LACS gene at 20 DAA; OsFAD2–1RNAi Line 22–4 (4) and Line 22–4 (5) were used as transgenic lines, *OsFAD2–1* RNAi Line 22–4 (1) and Line 22–4 (2) were used as the null segregants. All four lines were derived from one *OsFAD2–1* RNAi 22–4 T_2_ plant.* shows the significantly different at *P* < 0.05 levels

Long chain fatty acyl-CoA synthetases (LACS) are known to be involved in the breakdown of complex fatty acids. Among a total of five annotated *LACS* unigenes in rice, LOC_Os05g25310 was found to be significantly down regulated by -1.45 fold (*p* = 0.04) and -0.76 log2 fold in the HO line at 15 DAA (Table 3, Fig. 1a) compared to the null segregant. Such reduction of LOC_Os05g25310 was also verified by real time quantitative reverse transcriptase polymerase chain reaction (qRT-PCR) (Fig. 2) indicating the significant reduction of the expression at 15 DAA developmental stage. The significance of such a down-regulation remains unclear. There was no significant change in the expression of LOC_Os05g25310 at 10 and 20 DAA. Expression of LOC_Os05g25310 was the highest at 10 DAA with a gradual decrease as the rice grain development progressed.

### Effects on TAG assembly

As the major storage lipid in oilseeds, TAG is utilized to fuel seed germination and early seedling establishment prior to autotrophy by photosynthesis [30, 31]. Given the potential importance of the HO trait in rice bran oil, it is pivotal to understand whether and how the TAG biosynthesis, turnover and catabolism are impacted upon in the HO grains.

TAG biosynthesis starts with glycerol-3-phosphate (G3P). Apart from glycolysis, G3P could also be produced by the action of glycerol kinase (GK). There are 14 unigenes encoding for GK as annotated in the rice genome database [25]. None of the *GK* genes was affected in their expression in any of the time points in the HO rice line. Also, there was no effect on the expression of the 18 annotated genes encoding for GPAT required to form lysophosphatidic acid (LPA) at the next stage of TAG assembly.

LPA is acylated by a lysophosphatidic acid acyltransferase (LPAAT) enzyme to form phosphatidic acid (PA). Again the expression of annotated *LPAAT* genes (http://rice.plantbiology.msu.edu/) was not affected in the HO rice. Diacylglycerol (DAG) is generated by removing the phosphate group from PA by phosphatidic acid phosphohydrolase (PAP). *PAP1* (LOC_Os01g63060), *PAP2* (LOC_Os05g21180) and *PAP3* (LOC_Os05g37910) have been annotated in the rice genome database [25]. TAG can be synthesised from DAG in two ways, the acyl-CoA dependent which is normally known as the Kennedy pathway or the acyl-CoA independent pathway. DGAT catalyses the last step of Kennedy pathway by transferring an acyl group from acyl-CoA to DAG to generate *de novo* TAG and has been implicated as the key enzyme in determining the oil content in seed oil [32, 33]. Expression of *DGAT2* (LOC_Os06g22080) was found to increase with the seed development in the null segregant. At 15 DAA, expression of *DGAT2* was significantly down regulated by -1.71 fold (*p* = 7.73E-3) and -0.98 log2 fold (Table 3) in the HO line. There was no significant difference in the expression level of *DGAT2* gene at other time points between HO and the null segregant line (Table 3, Fig. 1b). DGAT2 has been regarded as a key enzyme in incorporation of unusual fatty acids such as epoxy or hydroxyl fatty acids in TAG to prevent their accumulation in the form of free fatty acids which might cause membrane dysfunction [34, 35]. The other DGAT enzyme, DGAT1, has low expression in the endosperm and no effect was detected.

The acyl-CoA independent reactions are involved in the conversion of two DAGs into a monoacyl glycerol (MAG) and a TAG by DAG:DAG transacylase [36, 37] or the conversion of DAG to TAG by an acyl transfer from the sn-2 position of PC to DAG by Phospholipid:diacylglycerolacyltransferase (PDAT) using PC as acyl donor in TAG formation [34, 38]. In the null segregant, among the 8 annotated PDAT unigenes, the majority of them were found to express at high levels at 10 DAA and decrease in expression in mature grains. Such an expression pattern was not affected in the HO line. The PDAT route is a mechanism for incorporation of unusual fatty acids in *Ricinus communis* by their direct transfer from PC to DAG [39, 40]. As unusual fatty acids have not been reported in rice bran oil, the significance of PDAT in RBO biosynthesis remains unresolved. The consistent expression between WT and HO rice may indicate the PDAT is not a key enzyme determining the oleic acid accumulation in RBO.

### Effect on genes involved in TAG packaging and oil body formation

TAG molecules synthesised are packaged and stored in oil bodies (OBs). OBs are maintained and protected by a single layer of PC and proteins which include oleosins, caleosins and steroleosins, with oleosin being the most abundant [41, 42]. Six oleosin genes, 9 caleosin genes and 1 steroleosin gene have been annotated in the rice genome database [25]. Our transcriptomics data showed that in the null segregant each of the three classes of oil body protein genes is expressed in all the three developmental stages examined, and increased as the grain developed. The expression of the oleosins was not found to be significantly affected in the HO line when compared to null segregant rice grain.

Caleosins are calcium- binding OB proteins. The expression of caleosins is reduced during germination to provide access to lipases for breakdown of TAG [53]. Among caleosins, the expression of LOC_Os02g50174 in the HO rice was significantly down regulated at both 15 and 20 DAA by -1.33 (and -0.63 log2 fold) and -1.97 fold (*p* = 0.04, 5.02E-3) (-0.97 log2 fold)respectively; (Table 3, Fig. 1b). Steroleosin has sterol-binding capacity and is mostly involved in signal transduction. The steroleosin unigene annotated as LOC_Os04g32080 was down regulated at 15 DAA by -1.36 fold (*p* = 0.03) and -0.65 log2 fold in the HO rice line (Table 3, Fig. 1b). It remains unclear how the down-regulation of *OsFAD2-1* in rice led to the down-regulation of OB protein gene expression. It is also of particular interest that such a change did not result in the reduction, but rather a modest increase of oil accumulation in HO rice.

### Effects on genes involved in fatty acid and lipid catabolism

The key genes coding for the enzymes involved in β-oxidation or fatty acid catabolism were also analysed. In general, all enoyl-CoA hydratase (ECH), 3- hydroxyacyl-CoA dehydrogenase (HACDH), ketoacyl-CoA thiolase (KAT) and acyl-CoA thioesterase (ACT) genes were expressed at high levels at 10 DAA and their expression level gradually decreased as seed development progressed. In the HO line, at 15 DAA stage the expression of ECH1 (LOC_Os01g70090) was significantly reduced by -1.64 fold (*p* = 0.03) and -0.93 log2 fold, compared to the null segregant (Table 3, Fig. 1a). Such reduction of the expression was also supported by qRT-PCR analysis (Fig. 2).

In the HO line, the majority of lipases are found to be expressed at high levels in the early developmental stage at 10 DAA and gradually decreased at later stages. Down-regulation of lipase promotes TAG stabilisation in rice [43]. Among all four phospholipases (PLC1-4), *PLC2* was found to be highly expressed with maximum expression at 10 DAA in null segregant. There was no significant variation on the PLC gene expression between the HO and null segregant.

### Expression of transcription factors that may be relevant to lipid accumulation

Apart from the genes that encode functional enzymes or proteins in the lipid biosynthesis or catabolism pathways, several transcription factors such as Leafy cotyledon1 (LEC1), LEC2 and FUSCA3 Like 1 (FL1), Wrinkled 1 (WRI1) and Abscisic acid-insensitive (ABI3) are also known to regulate fatty acid and TAG biosynthesis and play an important role in lipid accumulation in seed, in addition to their roles in seed development and maturation [44–49]. At 15 DAA, the expression level of the unigene LOC_Os02g49410 annotated as *LEC1* was significantly reduced by -1.66 fold (*p* = 3.91E-3) and -0.92 log2 fold in the HO line compared to the null segregant (Table 3, Additional file 5).

### Impact of *OsFAD2-1* RNAi down regulation on other genes

It was found that the expression of several genes not discussed above was also affected in the HO rice. These are not known to have a direct association with fatty acid and lipid biosynthesis (Additional file 7: Figure S1). For example, the expression of different storage protein genes were differentially regulated at all three stages in the HO rice grains (see Table 5). The expression patterns of additional selected genes being significantly affected in all the time points are also shown in Table 5. This data may facilitate the exploration of other potential molecular networks OsFAD2-1 might be involved, in addition to its key role in linoleic acid biosynthesis.

**Table 5:** Differential expression of non lipid genes between *OsFAD2-1*RNAi lines and their null segregant (NG)

Gene ID	Gene description	10 DAA (RPKM)				15 DAA (RPKM)				20 DAA (RPKM)			
		NG	RNAi	*p*-value	Fold change	NG	RNAi	*p*-value	Fold change	NG	RNAi	*p*-value	Fold change
LOC_Os05g26377	PROLM9 - precursor, expressed	10.42	34.97	3.33E-4	3.355	6.14	13.75	0.00	2.241	13.93	48.11	0.00	3.452
LOC_Os03g07226	Thioredoxin, putative, expressed	176.08	87.9	0.02	−2.00	234.84	134.73	2.16E-07	−1.743	431	74.28	0.00	−1.488
LOC_Os05g26770	PROLM18- precursor, expressed	144	391.26	5.56E-5	2.717	252.1	397.94	1.21E-05	1.578	783.1	2001.73	0.01	2.556
LOC_Os06g31070	PROLM24 precursor, expressed	7999.23	6629.43	0.03	−1.206	13109.13	8339.18	0.01	−1.571	21612.21	13605.77	0.01	−1.588
LOC_Os01g60410	Ubiquitinconjugating enzyme	392.22	271.47	0.02	−1.444	258.38	153.13	1.55E-05	−1.687	182.23	133.27	0.02	−1.367
LOC_Os03g55730	SSA2 - 2S albumin seed storage family protein precursor	7010.17	4731.88	4.97E-4	−1.481	7616.26	4233.57	0.01	−1.799	8507.59	5390.14	0.02	−1.578
LOC_Os05g33570	40S ribosomal protein S9-2	807.34	510.12	0.01	−1.582	402.52	183.09	5.65E-10	−2.198	99.06	61.26	0.04	−1.617

*DAA- *days after anthesis

## Conclusion

**Fig. 3:**
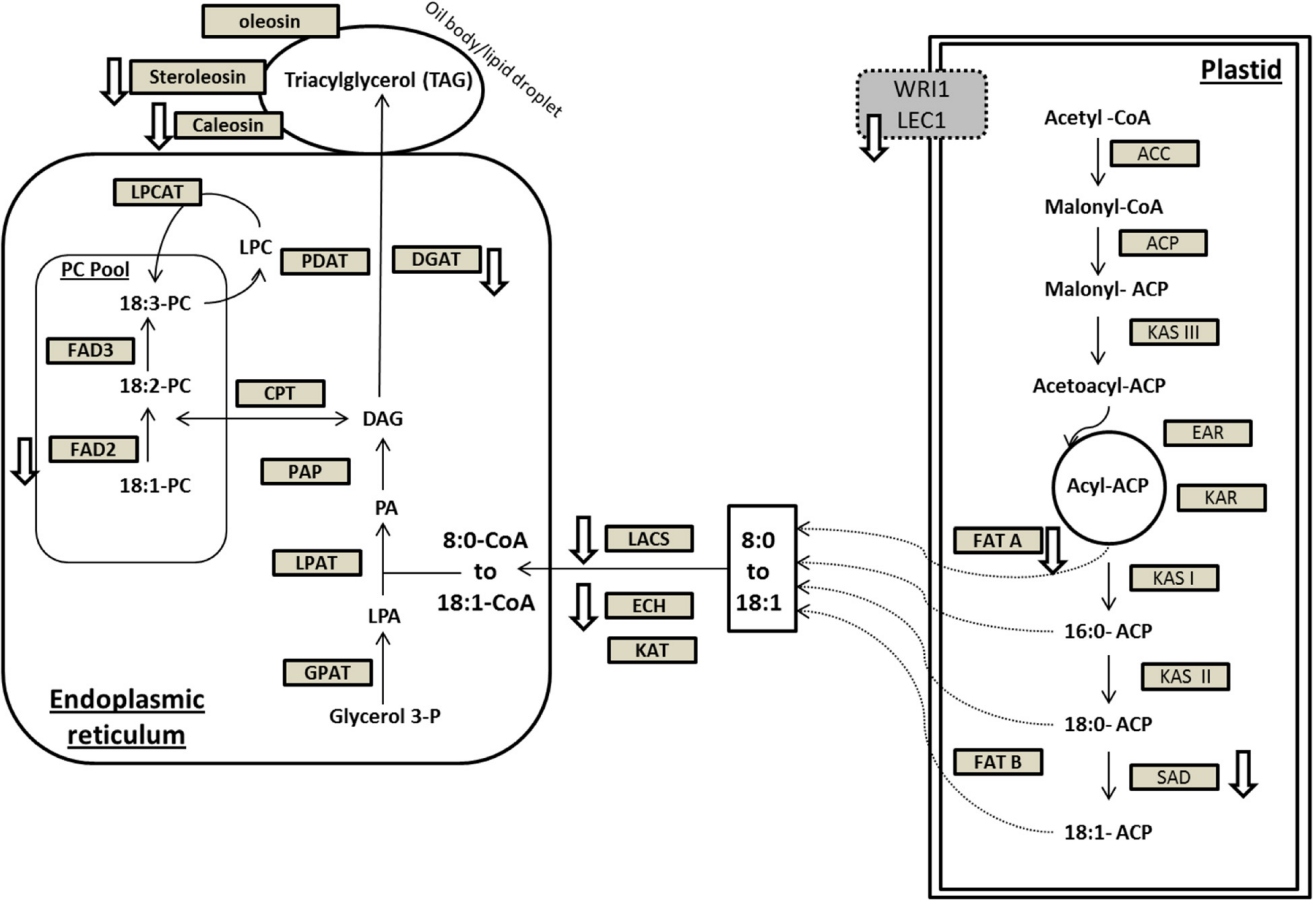
Differential expression of mRNAs of *OsFAD2-1* RNAi lines on genes involved in fatty acid biosynthesis and lipid metabolism. Downward arrows (↓) indicate down regulated the expression of specific genes in *OsFAD2-1* RNAi lines. ACC: acetyl-CoA carboxylase, ACP: Acyl carrier protein, KASIII: Beta-ketoacyl-ACP synthase III, EAR: Enoyl-ACP reductase, KAR: ketoacyl-ACP reductase, KASI: Beta-ketoacyl-ACP synthase I, KASII: Beta-ketoacyl-ACP synthase II, SAD: Stearoyl-ACP desaturase, FATA: Acyl-ACP thioesterase A, FATB: Acyl-ACP thioesterase B, LACS: Long-chain acyl-CoA synthetase, ECH: Enoyl-CoA hydratase, KAT: Ketoacyl-CoA Thiolase, GPAT: acyl-CoA:G3P acyltransferase, LPAT: acyl-CoA:LPAacyltransferase, PAP: PA phosphatase, CPT: CDP-choline:DAGcholinephosphotransferase, FAD: fatty acid desaturases, LPCAT: acyl-CoA:LPCacyltransferase, PDAT: phospholipid:DAGacyltransferase, DGAT: acyl-CoA:DAGacyltransferase, LPA: lyso-phosphatidic acid, PA: phosphatidic acid, DAG: diacylglycerol, PC: phosphatidylcholine, LEC1: Leafy cotyledon1, WRI1: Wrinkled 1

The transcriptomic analysis of the HO rice grains generated through RNAi down-regulation of *OsFAD2-1* suggests that a suite of key genes involved in fatty acid biosynthesis, TAG assembly and turnover have been differentially regulated in order to incorporate the increased level of oleic acid in TAG that is stored in the form of OBs. Further, the observation of a modest increase in TAG in the HO rice grains may also suggest that the availability of high level of oleic acid is likely favourable for TAG biosynthesis in rice. Overall, this study has delineated a subset of lipid-metabolism genes as being affected when *OsFAD2-1* is down-regulated and the proportion of oleic acid increases in TAG (Fig. 3). The impact on these genes is currently being verified by other techniques. It is envisaged that the genetic manipulation or co-expression of the genes clearly shown to be affected might lead to in further enhancement of the nutritionally desirable oleic acid and TAG accumulation in rice grains.

## Methods

### Plant materials

High oleic (HO) and null segregant rice (*O. sativa*cv. Nipponbare) seeds were harvested in CSIRO Agriculture, Australia where the HO rice line was previously developed [18]. One *OsFAD2-1*RNAi silencing line, *FAD2RNAi-22(4)* and a null segregant*, FAD2RNAi-22(8*) were used for this study. These were derived from the progeny from one single transformation event, *FAD2RNAi-22,* which had a dramatic reduction of the targeted gene expression and high level of oleic acid content [18]. Rice plants were grown in a containment glasshouse with a constant temperature regime of 27 °C (day and night) under natural light. Fifteen to twenty of immature seeds were collected at 10, 15 and 20 DAA respectively. The endosperms were isolated from the developing grains, frozen in liquid nitrogen and preserved at -80 °C freezer for RNA isolation. T_5_ seeds from T_4_ plants were analysed, whereas in Zaplin et al. [18], T_4_ seeds from T_3_ plants were analysed.

### Rice grain lipid analysis

Mature brown rice grains were obtained by manual de-hulling and ground with a CapMixTM capsule mixing device (3 M ESPE, Seefeld, Germany). Total lipids from ~300 mg above prepared rice flour samples were extracted with a mixture of chloroform/methanol/0.1 M KCl (at a ratio of 2/1/1, by volume). Fatty acid methyl esters (FAME) were prepared by incubating lipid samples in 1 N Methanolic-HCl (Supelco, Bellefonte, PA) at 80 °C for 2 h. TAG and polar membrane lipid pools were fractionated from total lipids in thin layer chromatography (TLC) (Silica gel 60, Merck, Darmstadt, Germany) using a solvent mixture of hexane/diethylether/acetic acid (at a ratio of 70/30/1, by volume) and individual membrane lipid classes were separated by TLC using a solvent mixture of chloroform/methanol/acetic acid/water (90/15/10/3, by volume). Authentic lipid standards were loaded and were run in separate lanes on the same plates for identification of lipid classes. Silica bands, containing individual class of lipid were used to prepare FAME as mentioned above and were analysed by gas chromatography GC-FID 7890A (Agilent Technologies, Palo Alto, CA) that was fitted with a 30 m BPX70 column (SGE, Austin, TX) for quantifying individual fatty acids on the basis of peak area of the known amount of heptadecanoin that was added in as an internal standard [50].

### RNA isolation and transcriptomic analysis

Total RNA was isolated from endosperm at 10, 15 and 20 DAA following the method of Higgins et al. [51] with modifications. For each RNA preparation, three endosperms were first ground in liquid nitrogen, then further ground with 600 μL NTES buffer (containing 100 mMNaCl, 10 mMTris, pH8.0, 1 mM EDTA and 1 % SDS), 800 μL phenol/chloroform (Sigma-Aldrich, St. Louis, MO). Samples were transferred into Eppendorf tubes and centrifuged at 13,000x rpm for 5 min in a microcentrifuge. After transferring into new Eppendorf tubes, the supernatant was mixed with an equal volume of 4 M LiCl/10 mM EDTA solution and kept at -20 °C overnight for RNA precipitation. RNA samples were precipitated by centrifugation at 10,000x rpm for 15 min at room temperature (25 °C), rinsed with 70 % ethanol and air dried. RNA pellets were dissolved in 360 μL milliQ H_2_O and 40 μL of 2 M NaOAc, pH5.8, which were then precipitated again with 1 mL 95 % ethanol and kept at -20 °C for 2 h. Samples were centrifuged, rinsed with 70 % ethanol and air dried as above. After drying, RNAs were dissolved in 20 μL DEPC water, and treated with RQ1 RNase-Free DNase (Promega, Madison, WI) following protocols. The quality of RNA samples were measured with Nanodrop 1000 Spectrophotometer for the ratios of OD 260 nm/280 nm (≥1.8) and OD260 nm/230 nm (≥1.8) and with Aligent Bioanalyser for RNA integrity number (RIN ≥ 6.5) score. RNA was normalised to 1 μg starting amounts in 50 μL. Sequencing libraries were prepared using the NEBNext® Ultra™ RNA Library Prep Kit for Illumina (New England Biolabs Inc., Ipswich, MA) following manufacturer’s instructions. Quantification and size estimation of libraries were performed on a Bioanalyser 2100 High Sensitivity DNA chip (Agilent Technologies, Waldbronn, Germany). Libraries were finally normalised to 2nM and sequenced on the Miseq System (Illumina Inc., San Diego, USA) generating 150 bp length single end reads.

### Transcriptomic analysis of *OsFAD2* genes from published databases

Six rice RNAseq libraries were down-loaded from Rice Gnome Annotation Project [25] that contains RNAseq databases from different tissues of *Nipponbare* rice. The RNAseq libraries were named SRR352184, 352187, 352190, 342204, 352206 and 352207 and derived from 20 day leaves, post-emergence inflorescence, anthers, 25 DAA embryo, 25 DAA endosperm and 10 DAA grain respectively. The read lengths were 40 or 35 bp and each run produced about 25 million ‘clean’ reads.

Four rice *FAD2* genes, *OsFAD2-1* to *OsFAD2-4*, were used as reference sequences to conduct gene mapping search “Map to Reference” against the databases in Additional file 6: Table S2 using a bioinformatic analysis program, Geneious [52]. Parameters used were set as custom sensitivity (for sensitivity), and none (fast/read mapping) (for Fine Tuning). Advanced settings were used with 10 % gap, 25 bp minimum overlap, 24 word length (words repeated more than 8 times were ignored), 2 % maximum mismatches per read, maximum gap 3, minimum overlap identity 80 %, index word length 14 and maximum ambiguity 4.

### Statistics analysis

Analysis of variation was performed using Genstat version 16 for lipid content and oleic acid content. All transcriptomics data of HO rice line and its null segregant was analysed using Qiagen CLC Genomics Workbench version 7.0.4. All statistical analysis was done using IBM SPSS Statistics version 20 and CLC Baggerley’s test (CLC Bio-Qiagen, Aarhus, Denmark). Details regarding the RNAseq analysis are available online athttp://www.clcbio.com/support/tutorials. For further verifying those differentially expressed genes determined by the method above, the read numbers for each cDNA were first converted to reads per kilo base per million (RPKM), then the ratios of RNAi and WT, and finally log2 value of the ratios.

### RNA extraction and quantitative real-time PCR (qRT-PCR)

Total RNA from endosperms at 10, 15 and 20 DAA was extracted using NucleoSpin®RNA Plant Kit (MachereyNagel, Duren, Germany) and quantified using Nanodrop1000 (Thermo Fisher Scientific, Waltham, MA). A total of 0.5 μg of RNA templates was used for the cDNA synthesis in a 50 μL reaction with ramp at 50 °C using Super Script III reverse transcriptase (Thermo Fisher Scientific). The cDNA template (100 ng) was used in a 10 μL qRT-PCR reaction with the annealing temperature at 58 °C. The primers for ECH1 gene were ECH1F(5′ GATGCTGGCGTTGCAAAGAT3′) and ECH1R (5′TCCCTGCTTCTCAGCAAAACA3′), for LACS gene were LACSF (5′TTGGCGAGGATGCACTGG 3’) and LACSR (5’TGGAACTGATTGCAGGTAGCTT 3’) which only amplified RT-PCR fragment in cDNAs. The primers for the *Tubulin* gene in rice were used as published [54]. The amplification was conducted in a Rotor-Gene 6000 (Corbett Life Sciences, Sydney, Australia) using Rotor Gene™ SYBR®Green PCR Kit (Qiagen, Hilden, Germany). Comparative quantification was analysed using *Tubulin* as a reference gene in the Real Time Rotary Analyzer Software (Corbett Life Sciences, Sydney, Australia). For each sample, triplicates of qRT-PCR reactions were performed.

## Additional files

Additional file 1: Amounts and fatty acid profiles of various lipids in Fad2 silenced rice. (XLSX 31 kb)
Additional file 2: List of unigenes involved in Fatty acid biosynthesis and catabolism. (XLSX 12 kb)
Additional file 3: List of unigenes involved in TAG acid biosynthesis and catabolism. (XLSX 14 kb)
Additional file 4: Table S1. (DOCX 17 kb)
Additional file 5: Gene expression (RPKM) values of affected genes in Lipid biosynthesis pathway. (XLSX 44 kb)
Additional file 6: Table S2. (DOC 30 kb)
Additional file 7: Figure S1. (DOCX 122 kb)
