# Supplementary material for: RNAi-mediated down-regulation of the expression of OsFAD2-1: effect on lipid accumulation and expression of lipid biosynthetic genes in the rice grain
Source: BMC Plant Biol. 2016 Aug 31;16:189. doi: 10.1186/s12870-016-0881-6 (PMC5007732; doi:10.1186/s12870-016-0881-6)
Supplement: Additional file 4: Table S1. — (DOCX 17 kb) [file 12870_2016_881_MOESM4_ESM.docx]

Supplementary Table 1. List of main enzymes involved in fatty acid biosynthesis and degradation

| Enzyme | Symbol | EC Number |
| --- | --- | --- |
| Fatty acid biosynthesis |  |  |
| Acetyl-CoA carboxylase | ACC | 6.4.1.2 |
| malonyl-CoA-ACP transacylase | MCMT | 2.3.1.3.9 |
| Beta-ketoacyl-ACP synthase I | KASI | 2.3.1.4.1 |
| Beta-ketoacyl-ACP synthase II | KASII | 2.3.1.179 |
| Beta-ketoacyl-ACP synthase III | KASIII | 2.3.1.180 |
| Enoyl-ACP reductase (NADH) | EAR | 1.3.1.9 |
| Acyl-ACP thioesterase A | Fat A | 3.1.2.14 |
| Acyl-ACP thioesterase B | Fat B | 3.1.2.14 |
| Fatty acid desaturation |  |  |
| ∆^12^ (ω6)-Desaturase | FAD | 1.14.19.6 |
| Stearoyl-ACP Desaturase | SAD | 1.14.19.2 |
| Fatty acid catabolism |  |  |
| Long-chain acyl-CoA synthetase | LACS | 6.2.1.3 |
| Acyl-CoA oxidase | ACX | 1.3.3.6 |
| Enoyl-CoA hydratase | ECH | 4.2.1.17 |
| 3s-hydroxyacyl-CoA dehydrogenase | HACDH | 1.1.1.35 |
| Ketoacyl-CoA Thiolase | KAT | 2.3.1.16 |
| Acyl-CoA Thioesterase | ACT | 3.1.2.2 |
| Enoyl-CoA isomerase | Isom | 5.3.3.8 |
|  |  |  |

Supplementary Table 2. Expression of four FAD2 genes in six different tissues from rice *Nipponbare* expressed as reads per million reads

| Gene name | RNAseq from 20 days leaves | RNAseq from post-emergence inflorescence | RNAseq from anther | RNAseq from 10 daa seed | RNAseq from 25 daa embryo | RNAseq from 25 daa endosperm |
| --- | --- | --- | --- | --- | --- | --- |
| FAD2-1 | 849 | 655 | 339 | 187 | 725 | 47 |
| FAD2-2 | 0 | 0 | 0 | 0 | 0 | 0 |
| FAD2-3 | 37 | 8.5 | 3 | 1 | 4 | 0 |
| FAD2-4 | 0 | 0 | 0 | 0 | 0 | 0 |

daa –days after anthesis
