# Supplementary material for: RNAi-mediated down-regulation of the expression of OsFAD2-1: effect on lipid accumulation and expression of lipid biosynthetic genes in the rice grain
Source: BMC Plant Biol. 2016 Aug 31;16:189. doi: 10.1186/s12870-016-0881-6 (PMC5007732; doi:10.1186/s12870-016-0881-6)
Supplement: Additional file 6: Table S2. — (DOC 30 kb) [file 12870_2016_881_MOESM6_ESM.doc]

Additional file 6: Table S2. Expression of four FAD2 genes in six different tissues from rice *Nipponbare* expressed as reads per million reads

| Gene name | RNAseq from 20 days leaves | RNAseq from post-emergence inflorescence | RNAseq from anther | RNAseq from 10 daa seed | RNAseq from 25 daa embryo | RNAseq from 25 daa endosperm |
| --- | --- | --- | --- | --- | --- | --- |
| FAD2-1 | 849 | 655 | 339 | 187 | 725 | 47 |
| FAD2-2 | 0 | 0 | 0 | 0 | 0 | 0 |
| FAD2-3 | 37 | 8.5 | 3 | 1 | 4 | 0 |
| FAD2-4 | 0 | 0 | 0 | 0 | 0 | 0 |

daa –days after anthesis
