# Supplementary material for: RNAi-mediated down-regulation of the expression of OsFAD2-1: effect on lipid accumulation and expression of lipid biosynthetic genes in the rice grain
Source: BMC Plant Biol. 2016 Aug 31;16:189. doi: 10.1186/s12870-016-0881-6 (PMC5007732; doi:10.1186/s12870-016-0881-6)
Supplement: Additional file 7: Figure S1. — (DOCX 122 kb) [file 12870_2016_881_MOESM7_ESM.docx]

**BB**


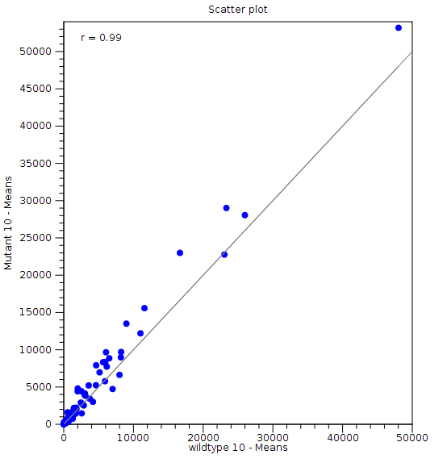

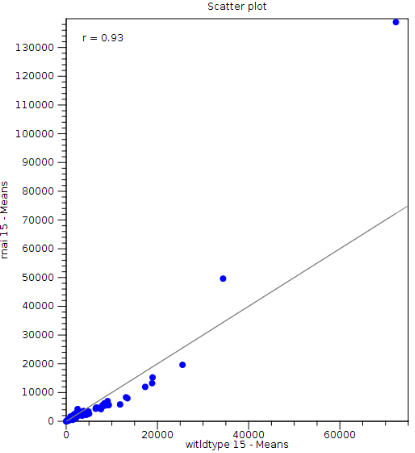


**A**


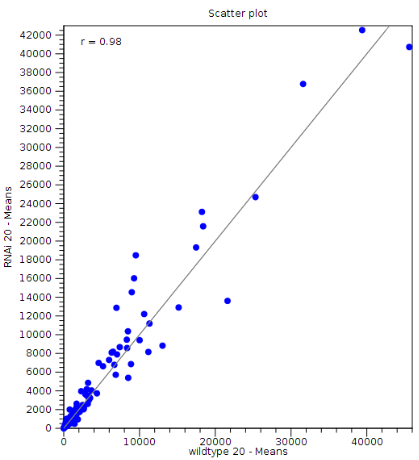


**CC**

**Figure 1. Scatter plot of the expression levels of all genes**. Comparison between null segregates (wild type) and *OsFAD2-1* RNAi lines at 10 daa (A), 15 daa (B) and 20 daa (C) stages. The values on the y-axis represent means of the expression levels of *OsFAD2-1* RNAi lines, the values on the x-axis represent the means of the expression levels of null segregates.
